# Adherence to sepsis protocol in a high-risk maternity reference center

**DOI:** 10.1590/0034-7167-2023-0453

**Published:** 2024-09-06

**Authors:** Marianny Medeiros de Moraes, Amuzza Aylla Pereira dos Santos, Juliana Duque da Silva de Sá Leitão, Kassiara Ferreira Felix de Lima Farias, Nathalya Anastácio dos Santos Silva, Núbia Vanessa da Silva Tavares, Kariane Omena Ramos Cavalcante de Melo, Isabel Comassetto

**Affiliations:** I Universidade Federal de Alagoas. Maceió, Alagoas, Brazil

**Keywords:** Pregnancy, Clinical Protocol, Maternity, Sepsis, Nursing, Embarazo, Protocolos Clínicos, Maternidades, Sepsis, Enfermería

## Abstract

**Objective:**

To describe the adherence to the sepsis protocol by obstetric nurses in the obstetric triage of a high-risk maternity reference center.

**Methods:**

This was a quantitative, documental, and retrospective study involving 105 pregnant women treated in obstetric triage under sepsis criteria. Data were collected through electronic medical records using structured forms and were organized into tables employing descriptive statistics. This research adhered to ethical principles concerning human studies.

**Results:**

Of the checklists for initiating the SEPSIS protocol by obstetric nurses, 105 were identified. Regarding the protocol steps performed, lactate was collected in 97.1% of cases and blood cultures in 98.1%, antibiotic therapy was administered in 94.3%, and hydration was carried out in 51.4% of the cases.

**Conclusion:**

The initiation of the sepsis protocol for all women meeting the criteria was confirmed. However, the steps were not fully implemented as recommended by the institutional protocol, and the recommended broad-spectrum antibiotic was not administered.

## RESUMEN

**Objetivo:**

Describir adhesión al protocolo de sepsis por enfermeras obstétricas en triaje obstétrico de una maternidad de referencia para embarazo de alto riesgo.

**Métodos:**

Estudio cuantitativo, documental y retrospectivo con 105 embarazadas atendidas en triaje obstétrico con critérios de sepsis. Realizada recolecta de datos mediante portuarios electrónicos utilizando formularios estructurados. Organizados los resultados en tablas, empleándose la estadística descriptiva. Esta investigación seguió los conceptos éticos que involucran estudios con seres humanos.

**Resultados:**

Identificados 105 *checklists* de apertura de protocolo de SEPSIS por enfermeras obstétricas. Cuanto a las etapas del protocolo de sepsis realizadas, recoletado lactato (97,1% de los casos) y hemocultura (98,1%), administrado antibioticoterapia (94,3%) y realizado hidratación (51,4%).

**Conclusion:**

Evidenciado apertura de protocolo de sepsis para todas las mujeres que rellenian los critérios. Aunque, las etapas no fueron efectuadas totalmente, como preconizado por protocolo institucional; y no fue administrado el antibiótico de amplio espectro recomendado.

## INTRODUCTION

Maternal sepsis is a life-threatening condition resulting from the body’s dysregulated response to infection during pregnancy, childbirth, or the postpartum period^(1)^. With a high fatality rate, it ranks among the top three causes of maternal mortality globally, accounting for approximately 260,000 deaths annually^(2)^.

The significant mortality rate is linked to the challenge of diagnosing sepsis because of physiological changes during pregnancy. Immunological and mechanical alterations, as well as adaptations such as tachycardia and hyperdynamic circulation, the effort exerted during the second stage of labor, interventions at birth, and blood loss can obscure signs of infection and hinder the early detection of sepsis^(3, 4)^.

From this perspective, delays in initiating treatment can escalate maternal mortality rates. A study in Mexico found that sepsis had the highest maternal mortality rate related to any specific disease, at 66%, followed by severe obstetric hemorrhage at 0.39%^(5)^. In the United States, 19.5% of pregnant women with confirmed sepsis progressed to septic shock, of which 14% died^(6)^.

A key strategy to mitigate adverse maternal outcomes is adherence to the sepsis protocol, which fosters organized actions in healthcare services to promptly recognize warning signs and implement timely interventions^(7)^. Recommended immediate measures upon suspecting maternal sepsis include lactate collection, obtaining blood cultures before administering antibiotics, administering broad-spectrum antibiotics, and, in cases of hypotension, fluid resuscitation with 30 ml/kg of crystalloid^(8, 9)^.

Therefore, employing protocols targeted at maternal sepsis is essential for improving quality, standardizing care, and fostering multidisciplinary collaboration^(4)^. In this context, the nursing professionals’ skills and competencies are critical in carefully assessing the patient and collaborating with the multidisciplinary team to ensure cohesive teamwork as per the sepsis protocol guidelines^(10)^.

It is necessary to validate the effectiveness of nursing practices— based on protocols for septic patients—and their applicability given the observed rates of maternal mortality and morbidity. This involves achieving the goals of global public health policies and the Sustainable Development Goals of the United Nations 2030 Agenda^(11)^.

Considering the severe repercussions and global impact of this condition, this study is justified by the need to understand how adherence to the sepsis protocol can reduce complications and prolonged hospitalization, which burden the Unified Health System (SUS), cause human losses, and undermine family health^(12)^.

This research is of significant scientific relevance as it highlights weaknesses in the care process and outlines strategies to enhance the quality of health team support. The goal is to improve the identification and management of this diagnosis, which is associated with a high rate of morbidity and mortality.

## OBJECTIVE

To describe the adherence to the sepsis protocol by obstetric nurses in the obstetric triage of a high-risk maternity reference center.

## METHODS

### Ethical aspects

This research adhered to the ethical principles outlined in Resolutions 466/12 and 510/16 of the National Health Council and was approved by the Research Ethics Committee. The data from this study are considered joint property of the involved parties. The information obtained concerning these patients was used solely for this research and did not identify the participants. Following these resolutions, a waiver was requested for the Informed Consent Form since data collection was performed using a database (medical records).

### Design, period, and location of study

This was a quantitative, documental, and retrospective study. The Strengthening the Reporting of Observational Studies in Epidemiology (STROBE) guidelines were followed for its description. Data collection began in August 2022 in the obstetric triage of a hospital recognized as a reference center for high-risk pregnancy within the Unified Health System (SUS), located in Recife, Pernambuco, Brazil.

### Population or sample; inclusion and exclusion criteria

The sample consisted of 105 pregnant patients treated in obstetric triage for whom a sepsis protocol was initiated. The analysis was non-probabilistic and of technical convenience. The sample included medical records where the protocol was initiated between January 2021 and July 2022. The timeframe was defined based on the month the institution implemented the protocol. All records that were incomplete or incorrectly filled out were excluded.

### Study protocol

Data collection was performed through electronic medical records between August 2022 and December 2022. The hospital’s computer center provided a list of patients who had the sepsis protocol checklist registered in their records. Obstetric nurses following the institutional protocol open these checklists in the patients’ electronic records. This data list included the patient’s name, medical record number, and the date and time the checklist was opened. Subsequently, patient data was collected.

A structured form, developed specifically for this purpose, was used as a tool for data collection. It covered clinical-epidemiological and demographic variables: age, race/color, education, origin, number of gestations, parities, gestational age, vital signs, infectious focus, MEOWS score, whether or not blood cultures were taken before administering intravenous antibiotic therapy, the time elapsed until administering antibiotics, admission to the Intensive Care Unit (ICU), and gestational outcomes.

### Data analysis and statistics

A análise de dados foi realizada mediante a construção de um banco de dados organizados e armazenados em uma planilha do software EXCEL (Microsoft Office) para a codificação das variáveis. Os resultados foram organizados em tabelas, utilizando-se a estatística descritiva.

## RESULTS

Of the checklists for initiating the SEPSIS protocol by obstetric nurses, 105 were identified. The sociodemographic profile of the pregnant women in the sample revealed that the majority were aged between 23 and 31 years (51%), self-identified as mixed race (68%), and were residents of Recife (43%). Most cases occurred in the third trimester of pregnancy (43%), followed by the second trimester (41%) and the first trimester (16%). Regarding parity, the majority were multiparous (64.8%).

In terms of signs and symptoms related to sepsis (Table 1), the primary complaints reported by the pregnant women at the time of risk classification included lower back pain (30.4%), lower abdominal pain (22.8%), dysuria (19.3%), increased body temperature (9.4%), vaginal bleeding (3.5%), and flank pain (3.5%).

**Table 1:** Frequency of main signs and symptoms related to maternal sepsis from January 2021 to July 2022. Recife, Pernambuco, Brazil, 2022

Main complaint	f	%
Chills	1	0.6
Dyspnea	1	0.6
Precordial Pain	1	0.6
Hematuria	1	0.6
Cough	2	1.2
Fluid Loss	3	1.7
Polyuria	4	2.3
Nausea and Vomiting	5	2.9
Flank Pain	6	3.5
Vaginal Bleeding	6	3.5
Increased Body Temperature	16	9.4
Dysuria	33	19.3
Lower Abdominal Pain	39	22.8
Lower Back Pain	52	30.4
Total	171	100

*f – frequency of symptoms*

Regarding the warning signs for sepsis (Table 2), among the vital signs measured in pregnant women meeting sepsis criteria, tachycardia was noted in 92.4% of cases, tachypnea in 20.9%, fever in 20.9%, and hypotension (with systolic blood pressure below 90 mmHg) in 10.5%.

**Table 2:** Vital signs measured in pregnant women with sepsis criteria from January 2021 to July 2022. Recife, Pernambuco, Bazil, 2022

Vital Signs	n	%
Blood pressure		
Hypotensive	11	10.5
Normotensive	80	76.1
Hypertensive	14	13.3
Total	105	100
Heart rate		
Normal Heart Rate	8	7.6
Tachycardia	97	92.4
Total	105	100
Respiratory rate		
Eupnea	77	73.3
Tachypnea	22	20.9
No Record	6	5.7
Total	105	100
Axillary temperature		
Afebrile	78	74.3
Febrile	22	20.9
No Record	5	4.8
Total	105	100

When analyzing the suspected source of sepsis (Table 3), the urinary tract was identified as the primary source in 87% of cases, followed by infection because of premature rupture of the ovular membranes (6%), infected abortion (5%), and pulmonary infection (3%).

**Table 3:** Main infectious foci in pregnant women with sepsis criteria from January 2021 to July 2022. Recife, Pernambuco, Brazil, 2022

Main Complaint	n	%
Pulmonary Infection	3	3%
Infected Abortion	5	5%
PROM	6	6%
Urinary	91	87%
Total	105	100

*PROM – Premature rupture of ovular membranes*

As for implementing the sepsis protocol steps (Table 4), lactate collection occurred in 97.1% of cases, and blood cultures were taken in 98.1% of cases. Antibiotic therapy was administered in 94.3% of cases, and hydration was performed in 51.4% of cases. Regarding the antibiotics administered (Table 5), ceftriaxone was prescribed in 90% of the cases, following protocol recommendations, accompanied by ampicillin (4%), cefalotin (3%), a combination of clindamycin and gentamicin (2%), and oral cephalexin (1%). Obstetric medical professionals prescribed antibiotics not included in the protocol, suspending the institutional protocol steps that the nursing team had initiated, based on the clinical presentation of the patients.

**Table 4:** Adherence to the maternal sepsis protocol from January 2021 to July 2022. Recife, Pernambuco, Brazil, 2022

Protocol steps	n	%
Lactate collection		
Yes	102	97.1
No	3	2.9
Total	105	100
Blood culture collection		
Yes	103	98.1
No	2	1.9
Total	105	100
ATB within one hour		
Yes	99	94.3
No	6	5.7
Total	105	100
Hydration		
Yes	54	51.4
No	51	48.6
Total	105	100

*ATB – antibiotics.*

## DISCUSSION

Sepsis has become a significant cause of maternal morbidity and mortality, with obstetric and neonatal consequences. In developed countries, adherence to well-established protocols and guidelines has had a substantial impact on reducing deaths caused by these complications^(13)^.

The data indicated that the study population primarily consisted of pregnant women in their third trimester, self-identified as mixed race, young, from the capital of Pernambuco, and multiparous. Contrasting with the finding related to gestational age, a study conducted in a hospital in Curitiba, state of Paraná, observed a higher prevalence of pregnant women admitted with sepsis in the second trimester of pregnancy; however, it showed that the association between “gestational age” and “severity of sepsis” was not statistically significant^(14)^.

Regarding signs and symptoms, the main complaints reported were increased body temperature, dysuria, lower abdominal pain, and lower back pain. As for the vital signs measured at risk classification, a significant percentage of patients exhibited hypertension, tachycardia, tachypnea, and fever. These findings are supported by a study that evaluated cases of maternal sepsis treated at a hospital in Campinas-SP, which demonstrated that the most prevalent symptoms were fever, lower back pain, and polyuria/dysuria^(15)^.

As for the infectious focus, there was a higher incidence of sepsis resulting from the urinary tract. Such condition stems from physiological changes during pregnancy that affect the urinary system. These changes significantly contribute to this finding because, during this period, there is a relaxation of the smooth musculature, extrinsic compression by the gravid uterus, increased urinary output, and immunological changes leading to greater urinary stasis. Consequently, urinary infections are very common during pregnancy and may be associated with adverse perinatal outcomes^(16)^.

In terms of adherence to the protocol and the timely interval between detection and intervention, the results demonstrated that in most cases, actions were appropriately taken within an hour. These findings are in line with the guidelines of the Surviving Sepsis Campaign (SSC), which recommend that treatment should be initiated within the first hour, also referred to as the “golden hour”^(8)^.

Therefore, nursing team must apply the sepsis protocol with the primary objective of accelerating the detection of disease signs and the initiation of protocol measures. This urgency is crucial because these professionals are close to the patient from the entrance of health services to the bedside^(16)^. The effectiveness of these actions can directly influence the reduction of maternal morbidity and mortality and its implications, such as increased lengths of hospital stays and associated costs^(4)^.

About developing the protocol steps, the results indicated a fragmentation of therapeutic care actions and continuity of care. According to Veras^(17)^, the steps of the sepsis protocol should not be fragmented but must be continuous, as these components form a cohesive whole and are designed to expedite therapeutic and care actions for obstetric patients to achieve positive outcomes.

The steps least properly implemented were adherence to antibiotic administration and the failure to use medications recommended by the protocol. Administering crystalloids is indicated only for hypotensive patients, which justifies the fewer instances it was performed. Research indicates that delays in administering the appropriate antibiotics increase the risk of death in sepsis cases^(18, 19, 20)^. A study conducted in Western China on bacterial sepsis demonstrated that the early initiation of appropriate antibiotic treatment enhances its effectiveness^(18)^.

The justification for fragmenting the protocol steps, as noted in the progress records, was a lack of integration within the multidisciplinary team, particularly concerning the prescription of antibiotics, which were, in some cases, altered or suspended by medical staff.

This observation supports findings from a study in the obstetric sector of a hospital in Northeastern Brazil, which identified issues in communication among professional groups and in the roles they play in managing sepsis, directly influencing the appropriate treatment for patients^(4)^.

From this perspective, Chaaban^(21)^ emphasizes the need to clarify the professional roles within the health team, improve communication between categories, and enhance the autonomy of obstetric nursing in managing cases according to the institutional protocol. The objective is to minimize errors in both the identification and diagnosis of sepsis and in the implementation of treatment measures.

### Study Limitations

Identifying which medical records had initiated the sepsis protocol proved challenging, as the records lacked the names of patients who underwent the protocol stages. The solution involved utilizing IT technicians who could identify the records through the protocol checklist registered in the system. Additionally, there was a notable lack of epidemiological data on the patients, especially concerning marital status, education, and occupation.

### Contributions to the Field

This study encourages the enhancement of nursing autonomy in practices governed by protocols for suspected or confirmed sepsis cases. The nursing professional is integral from the entry point of health services, through risk classification, to bedside care in the hospitalization process. This continuous presence enhances the early identification of sepsis cases, thereby preventing unfavorable outcomes.

## CONCLUSION

The data indicate that a significant proportion of pregnant women exhibited alterations in vital signs such as hypertension, tachycardia, tachypnea, and fever. The primary source of infection in the sepsis cases was the urinary tract. A sepsis protocol was initiated for all patients who met the criteria; however, the steps were not fully executed as per the institutional guidelines. Furthermore, the recommended broad-spectrum antibiotics were not administered because of the healthcare professionals’ unfamiliarity with the protocol stages.

Therefore, it is essential to enhance awareness about sepsis through continuous education for the healthcare team. Continual internal audits of patient records are also necessary to assess and improve compliance with the protocols associated with this clinical condition, which significantly affects public health.
