# Supplementary material for: Adherence to sepsis protocol in a high-risk maternity reference center
Source: Rev Bras Enferm. 2024 Sep 6;77(4):e20230453. doi: 10.1590/0034-7167-2023-0453 (PMC11382675; doi:10.1590/0034-7167-2023-0453)
Supplement: Translation

# Translation: Adesão ao protocolo de sepse em uma maternidade de referência para alto risco

**DOI:** 10.1590/0034-7167-2023-0453pt

**Authors:** Marianny Medeiros de Moraes, Amuzza Aylla Pereira dos Santos, Juliana Duque da Silva de Sá Leitão, Kassiara Ferreira Felix de Lima Farias, Nathalya Anastácio dos Santos Silva, Núbia Vanessa da Silva Tavares, Kariane Omena Ramos Cavalcante de Melo, Isabel Comassetto

**Affiliations:** I Universidade Federal de Alagoas. Maceió, Alagoas, Brasil

**Keywords:** Gravidez, Protocolo Clínico, Maternidade, Sepse, Enfermagem

## Abstract

**Objetivo:**

Descrever a adesão ao protocolo de sepse por enfermeiras obstétricas na triagem obstétrica de uma maternidade de referência para gestação de alto risco.

**Métodos:**

Estudo quantitativo, documental e retrospectivo com 105 gestantes atendidas na triagem obstétrica com critérios de sepse. Realizou-se coleta de dados mediante prontuários eletrônicos utilizando formulários estruturados. Organizaram-se os resultados em tabelas, empregando-se a estatística descritiva. Esta pesquisa seguiu os conceitos éticos que envolvem estudos com seres humanos.

**Resultados:**

Identificaram-se 105 *checklists* de abertura de protocolo de SEPSE por enfermeiras obstétricas. Quanto às etapas do protocolo de sepse realizadas, coletaram-se lactato (97,1% dos casos) e hemocultura (98,1%), administrou-se antibioticoterapia (94,3%) e realizou-se hidratação (51,4%).

**Conclusão:**

Evidenciou-se abertura de protocolo de sepse para todas as mulheres que preenchiam os critérios. No entanto, as etapas não foram efetivadas totalmente como preconizado pelo protocolo institucional; e não foi administrado o antibiótico de largo espectro recomendado.

## INTRODUÇÃO

A sepse materna é uma condição de risco de vida decorrente das respostas desreguladas do organismo à infecção durante a gestação, parto ou puerpério^(1)^. Em razão de seu alto índice de letalidade, está entre as três maiores causas de mortalidade materna no mundo, sendo responsável por cerca de 260 mil mortes por ano^(2)^.

A ocorrência da alta mortalidade está relacionada ao difícil diagnóstico devido às alterações fisiológicas que ocorrem durante a gravidez. As modificações imunológicas e mecânicas, as adaptações da gravidez como taquicardia e circulação hiperdinâmica, esforços maternos no segundo estágio do parto, intervenções no parto e perdas sanguíneas podem mascarar sinais de infecção e dificultar a identificação precoce da sepse^(3, 4)^.

Sob essa perspectiva, o atraso no início das intervenções pode aumentar os índices de mortalidade materna. Estudo realizado no México relatou que a sepse apresentou a maior taxa de mortalidade materna relacionada à doença específica, com 6,6%, seguida pela hemorragia obstétrica grave, com 0,39%^(5)^. Nos Estados Unidos, 19,5% das gestantes com sepse confirmada evoluíram para choque séptico, das quais 1,4% evoluiu para óbito^(6)^.

Diante desse contexto, uma estratégia proposta para redução dos desfechos maternos desfavoráveis é a adesão ao protocolo de sepse, que conduz a uma ação organizada nos serviços de saúde para reconhecer precocemente os sinais de alerta, conduzindo a intervenções em tempo oportuno^(7)^. Para tanto, preconizam-se condutas a serem tomadas na primeira hora após a suspeita de sepse materna: coleta de lactato, obtenção de culturas antes de começar o antibiótico, administração de antibióticos de amplo espectro; e, em casos de hipotensão, ressuscitação volêmica com 30 ml/kg de cristaloide^(8, 9)^.

Assim, a utilização de protocolos direcionados à sepse materna é crucial para oferecer melhoria da qualidade, padronizar os cuidados e estabelecer clima de colaboração multidisciplinar^(4)^. Nesse contexto, as habilidades e competências do profissional de enfermagem acompanham a importante responsabilidade de avaliar criteriosamente a paciente e participar do processo de tomada de decisões com a equipe multiprofissional, para garantir o trabalho em equipe, de acordo com as recomendações do protocolo de sepse^(10)^.

Destarte, é necessário validar tanto a importância da prática pela equipe de enfermagem — com base em protocolos para pacientes sépticos — quanto sua aplicabilidade diante dos índices de mortalidade e morbidade materna observados. Isso significa alcançar as metas das políticas públicas mundiais de saúde materna e dos Objetivos de Desenvolvimento Sustentável da Agenda 2030 das Nações Unidas^(11)^.

Em face das repercussões negativas e impacto global desse fenômeno, este estudo justifica-se pela necessidade de conhecer como a adesão ao protocolo de sepse pode contribuir para reduzir os agravos e prolongamento da internação, que elevam o ônus do SUS, geram perdas humanas e diminuem a saúde da família^(12)^.

Este estudo é de grande relevância científica por revelar as fragilidades no processo de cuidado e nortear estratégias para melhorar a qualidade da assistência pela equipe de saúde. Com isso, a finalidade é melhorar a identificação e manejo desse diagnóstico, por trás do qual está uma doença com grande índice de morbimortalidade.

## OBJETIVO

Descrever a adesão ao protocolo de sepse por enfermeiras obstétricas na triagem obstétrica de uma maternidade de referência para gestação de alto risco.

## MÉTODOS

### Aspectos éticos

Esta pesquisa seguiu os conceitos éticos das Resoluções 466/12 e 510/16, do Conselho Nacional de Saúde, sendo aprovado pelo Comitê de Ética em Pesquisa. Os dados do estudo em questão são considerados propriedade conjunta das partes envolvidas. As informações obtidas relacionadas a essas pacientes foram utilizadas única e exclusivamente nesta pesquisa, sem identificar as participantes.

Em consonância com as resoluções, foi solicitada a dispensa do Termo de Consentimento Livre e Esclarecido, uma vez que a coleta de dados da referida pesquisa foi realizada em banco de dados (prontuários).

### Desenho, período e local do estudo

Trata-se de um estudo quantitativo, documental e retrospectivo. Para sua descrição, foram seguidas as recomendações das diretrizes *Strengthening the Reporting of Observational Studies in Epidemiology* (STROBE).

A coleta de dados foi iniciada em agosto de 2022 na triagem obstétrica de um hospital de referência para gestação de alto risco, pertencente ao Sistema Único de Saúde (SUS), localizado na cidade de Recife, estado de Pernambuco, Brasil.

### População ou amostra; critérios de inclusão e exclusão

A amostra foi composta por 105 pacientes gestantes atendidas na triagem obstétrica para as quais foi aberto protocolo de sepse. A análise foi não probabilística e de conveniência técnica de amostragem. Foram incluídos na amostra os prontuários de pacientes nos quais o referido protocolo foi aberto entre janeiro de 2021 e julho de 2022. O recorte de tempo foi definido a partir do mês em que houve a implementação do protocolo na instituição. Foram excluídos todos os prontuários que apresentassem informações incompletas e preenchidos de forma incorreta.

### Protocolo do estudo

A coleta de dados foi realizada por meio de prontuários eletrônicos, entre agosto de 2022 e dezembro de 2022. Para tanto, foi solicitado ao centro de informática hospitalar a relação das pacientes que tinham o registro do *checklist* do protocolo de sepse no prontuário. Os *checklists* são abertos no prontuário eletrônico da paciente por enfermeiros obstétricos conforme o protocolo institucional. Essa lista de dados continha o nome da paciente, número de registro de prontuário e a data e hora em que foi aberto o *checklist.* Posteriormente foi realizado o levantamento dos dados das pacientes.

Foi utilizado como instrumento para a coleta de dados um formulário estruturado elaborado para esse fim, que contemplou as variáveis clínico-epidemiológicas e demográficas: idade, raça/cor, escolaridade, procedência, gestas, paridades, idade gestacional, sinais vitais, foco infeccioso, escore de MEOWS, coleta ou não das hemoculturas antes da administração da antibioticoterapia intravenosa, tempo decorrido até a administração do antibiótico no paciente, internamento em Unidade de Terapia Intensiva (UTI) e desfechos gestacionais.

### Análise de dados e estatística

A análise de dados foi realizada mediante a construção de um banco de dados organizados e armazenados em uma planilha do software EXCEL (Microsoft Office) para a codificação das variáveis. Os resultados foram organizados em tabelas, utilizando-se a estatística descritiva.

## RESULTADOS

Foram identificados 105 *checklists* de abertura de protocolo de SEPSE por enfermeiras obstétricas. Quanto à caracterização sociodemográfica das gestantes que compõem a amostra, a maioria tinha idade entre 23 e 31 anos (51%), autodeclaravam-se pardas (68%) e eram provenientes da cidade de Recife (43%). No tocante à idade gestacional no período da admissão, a maioria dos casos ocorreram no terceiro trimestre (43%), seguido do segundo trimestre (41%) e primeiro trimestre (16%). Quanto à paridade, a maioria eram multíparas (64,8%).

No que se refere aos sinais e sintomas relacionados à sepse (Tabela 1), as principais queixas referidas pelas gestantes no momento da classificação de risco foram dor lombar (30,4%), dor em baixo ventre (22,8%), disúria (19,3%), aumento da temperatura corporal (9,4%), sangramento vaginal (3,5%) e dor em flanco (3,5%).

**Tabela 1:** Frequência dos principais sinais e sintomas relacionados à sepse materna entre janeiro de 2021 e julho de 2022. Recife, Pernambuco, Brasil, 2022

Queixa principal	f	%
Calafrio	1	0,6
Dispneia	1	0,6
Dor precordial	1	0,6
Hematúria	1	0,6
Tosse	2	1,2
Perdas líquidas	3	1,7
Polaciúria	4	2,3
Náuseas e vômito	5	2,9
Dor em flanco	6	3,5
Sangramento vaginal	6	3,5
Aumento da temperatura corporal	16	9,4
Disúria	33	19,3
Dor em baixo ventre	39	22,8
Dor lombar	52	30,4
Total	171	100

*f – frequência dos sintomas*

Quanto aos sinais de alerta para sepse (Tabela 2), inerentes aos sinais vitais aferidos nas gestantes com critério de sepse, apresentou-se taquicardia (92,4%), taquipneia (20,9%), febre (20,9%) e hipotensão com pressão sistólica abaixo de 90 mmHg (10,5%).

**Tabela 2:** Sinais vitais aferidos em gestantes com critério de sepse entre janeiro de 2021 e julho de 2022. Recife, Pernambuco, Brasil, 2022

Sinais vitais	n	%
Pressão arterial		
Hipotenso	11	10,5
Normotenso	80	76,1
Hipertenso	14	13,3
Total	105	100
Frequência cardíaca		
Normocardia	8	7,6
Taquicardia	97	92,4
Total	105	100
Frequência respiratória		
Eupneia	77	73,3
Taquipneia	22	20,9
Sem registro	6	5,7
Total	105	100
Temperatura axilar		
Afebril	78	74,3
Febril	22	20,9
Sem registro	5	4,8
Total	105	100

Ao analisar o foco sugestivo de origem da sepse (Tabela 3), o urinário foi o principal, representando 87%, seguido de infecção decorrente da ruptura prematura das membranas ovulares (6%), aborto infectado (5%) e infecção pulmonar (3%).

**Tabela 3:** Principais focos infecciosos em gestante com critério de sepse, entre janeiro de 2021 a julho de 2022. Recife, Pernambuco, Brasil, 2022

Queixa principal	n	%
Infecção pulmonar	3	3%
Aborto infectado	5	5%
RPMO	6	6%
Urinário	91	87%
Total	105	100

*RPMO – ruptura prematura das membranas nas ovulares.*

Quanto às etapas do protocolo de sepse realizadas (Tabela 4), coletaram-se lactato (em 97,1% dos casos) e hemocultura (98,1%), administrou-se antibioticoterapia (94,3%) e realizou-se hidratação (51,4%). Dos antibióticos administrados (Tabela 5), em 90% dos casos foram prescritos ceftriaxona conforme a recomendação do protocolo, seguido de ampicilina (4%), cefalotina (3%), clindamicina associado a gentamicina (2%) e cefalexina oral (1%). Os antibióticos administrados não instituídos no protocolo foram prescritos por profissionais médicos obstetras, que suspenderam as etapas do protocolo institucional iniciadas pela equipe de enfermagem e basearam-se na clínica da paciente.

**Tabela 4:** Adesão ao protocolo de sepse materna entre janeiro de 2021 a julho de 2022. Recife, Pernambuco, Brasil, 2022

Etapas do protocolo	n	%
Coleta de lactato		
Sim	102	97,1
Não	3	2,9
Total	105	100
Coleta de hemocultura		
Sim	103	98,1
Não	2	1,9
Total	105	100
ATB até uma hora		
Sim	99	94,3
Não	6	5,7
Total	105	100
Hidratação		
Sim	54	51,4
Não	51	48,6
Total	105	100

*ATB – Antibiótico.*

## DISCUSSÃO

A sepse tornou-se uma causa importante de morbimortalidade materna, com consequências obstétricas e neonatais. Nos países desenvolvidos, a adesão a protocolos e diretrizes bem estabelecidos resultou em maior impacto na redução de óbitos maternos decorrentes de complicações ocasionadas por esse agravo^(13)^.

Os dados demonstraram que a população deste estudo é composta, em sua maioria, por gestantes que estavam no terceiro trimestre de gestação, autodeclaradas pardas, jovens, provenientes da capital pernambucana e multíparas. Divergindo do achado referente à idade gestacional, estudo realizado em um hospital de Curitiba, estado do Paraná, obteve maior prevalência de gestantes admitidas com sepse no segundo trimestre de gestação, no entanto demonstrou que a associação das variáveis “idade gestacional” e “gravidade da sepse” não foi estatisticamente significante^(14)^.

No que se refere aos sinais e sintomas, as queixas principais mais relatadas foram aumento da temperatura corporal, disúria, dor em baixo ventre e dor lombar. Quanto aos sinais vitais aferidos na classificação de risco, um percentual significativo apresentou hipertensão, taquicardia, taquipneia e febre. Esses achados corroboram um estudo que avaliou casos de sepse materna atendidos em um Hospital de Campinas-SP e evidenciou que os sintomas mais prevalentes foram febre, dor lombar e polaciúria/disúria^(15)^.

Quanto ao foco infeccioso, obteve-se maior incidência de sepse decorrente do foco urinário. Tal condição advém das alterações fisiológicas gravídicas sobre o trato urinário. Essas mudanças exercem importante influência sobre tal achado porque, nesse período, ocorre relaxamento da musculatura fina, compressão extrínseca pelo útero gravídico, aumento do débito urinário e alterações imunológicas, levando a maior estase urinária. Por isso, as infecções urinárias são muito comuns durante a gestação e podem estar associadas com desfecho perinatal adverso^(16)^.

No tocante à adesão ao protocolo e tempo oportuno entre a detecção e realização das intervenções, os resultados demonstraram que, em sua maioria, ocorreram adequadamente em uma hora. Esses achados alinham-se com as diretrizes da *Surviving Sepsis Campaign* (SSC), em que o tratamento deve ser instituído na primeira hora, também denominada “hora de ouro”^(8)^.

Desse modo, compreende-se que a equipe de enfermagem deve aplicar o protocolo de sepse com o objetivo primordial de agilizar a detecção de sinais da doença e início das medidas do protocolo. Isso porque são esses profissionais que se encontram próximos ao paciente desde a porta de entrada dos serviços de saúde até a beira do leito^(16)^. A efetividade dessas ações pode influenciar diretamente a redução da morbimortalidade materna e suas implicações, como tempo aumentado de internamento e de custos de hospitalidade^(4)^.

Sobre o desenvolvimento das etapas do protocolo, os resultados mostraram haver fragmentação de ações assistenciais, terapêuticas e na linha de cuidados. Para Veras^(17)^, as etapas do protocolo de sepse não podem ser fragmentadas, mas contínuas, já que essas partes constituem o todo, logo têm o objetivo comum de agilizar as ações terapêuticas e de cuidado da paciente obstétrica para gerar resultados positivos.

As etapas do protocolo menos instituídas adequadamente foram a adesão à administração do antibiótico e a não utilização da medicação recomendada pelo protocolo. A etapa da administração de cristaloide está indicada apenas para pacientes hipotensos, o que justifica o número reduzido na realização.

Destarte, estudos mostram que o atraso no uso do antibiótico adequado aumenta o risco de morte em caso de sepse^(18, 19, 20)^. Nessa linha, uma pesquisa realizada no Oeste da China sobre sepse bacteriana evidenciou que o início precoce da administração de antibiótico adequado favorece a eficácia do tratamento^(18)^.

No que tange à justificativa da fragmentação das etapas do protocolo, foi identificado nas evoluções encontradas nos prontuários que houve a falta de integração da equipe multipro-fissional nas condutas, principalmente no tocante à prescrição de antibiótico, os quais, em alguns casos, foram substituídos ou suspensos pela categoria médica.

Esse achado corrobora um estudo desenvolvido no setor obstétrico de um hospital do Nordeste brasileiro. Os pontos a serem melhorados foram os problemas na comunicação entre as categorias profissionais e nos papéis desempenhados diante do quadro sepse, pois influenciam diretamente o tratamento adequado para paciente^(4)^.

Sob essa perspectiva, Chaaban^(21)^ demonstra a necessidade de abordar os papéis profissionais desempenhados pela equipe de saúde, melhorar a comunicação entre as categorias e fortalecer a autonomia da enfermagem obstétrica na condução dos casos conforme protocolo institucional. O objetivo é diminuir erros tanto de identificação e de diagnósticos quanto nas ações do pacote de medidas.

### Limitações do Estudo

Houve dificuldade para identificar em quais prontuários foi aberto o protocolo de sepse, pois não tinham registros com os nomes das pacientes que passaram pelas etapas do protocolo. A estratégia encontrada para listar o nome das pacientes foi recorrer aos técnicos de informática, que conseguiram identificar os prontuários por meio do *checklist* do protocolo registrado no sistema. Além disso, notou-se carência de dados epidemiológicos da paciente, principalmente quanto a estado civil, escolaridade e ocupação.

### Contribuições para a Área

O presente estudo estimula o fortalecimento da autonomia da enfermagem em condutas estabelecidas por protocolo aplicado aos quadros de sepse suspeitos ou confirmados. Isso porque o profissional de enfermagem encontra-se desde a porta de entrada dos serviços de saúde, passando pela classificação de risco, até a beira do leito no processo de internação. Esse acompanhamento favorece a identificação precoce dos quadros de sepse, evitando desfechos desfavoráveis.

## CONCLUSÃO

Os dados mostram que um percentual considerável das gestantes apresentou alteração dos sinais vitais, como hipertensão, taquicardia, taquipneia e febre. O principal foco infeccioso do quadro de sepse foi o urinário. Foi aberto protocolo de sepse para todas as pacientes que preenchiam os critérios. No entanto, as etapas não foram efetivadas totalmente como preconizado pelo protocolo institucional. Além disso, não foi administrado o antibiótico de largo espectro recomendado, pois os profissionais que atendem nessa instituição não conheciam as etapas do protocolo.

Dessa forma, é necessário um processo de sensibilização quanto à sepse mediante a educação permanente da equipe acerca da temática. Também devem ser implementadas auditorias internas contínuas dos prontuários dos pacientes para avaliar e melhorar o processo de adesão às condutas referentes a essa condição clínica, a qual tem forte impacto na saúde pública.
